# Prevalence of DSM-5 mental disorders in a nationally representative sample of children in Taiwan: methodology and main findings

**DOI:** 10.1017/S2045796018000793

**Published:** 2019-01-30

**Authors:** Yi-Lung Chen, Wei J. Chen, Kuan-Chia Lin, Lih-Jong Shen, Susan Shur-Fen Gau

**Affiliations:** 1 Department of Psychiatry, National Taiwan University Hospital and College of Medicine, Taiwan; 2 Institute of Epidemiology and Preventive Medicine, College of Public Health, National Taiwan University, Taiwan; 3 Genetic Epidemiology Core, Center of Genomic Medicine, National Taiwan University, Taiwan; 4 Department of Health Care Management, National Taipei University of Nursing and Health Sciences, Taiwan; 5 Department of Mental and Oral Health, Ministry of Health and Welfare, Taiwan; 6 Graduate Institute of Brain and Mind Sciences and Institute of Clinical Medicine, College of Medicine, National Taiwan University, Taiwan

**Keywords:** Children, DSM-5, epidemiology, national survey, prevalence

## Abstract

**Aims:**

There has been a lack of prevalence estimates of DSM-5 mental disorders in child populations at the national level worldwide. This study estimated the lifetime and 6-month prevalence of mental disorders according to the DSM-5 diagnostic criteria in Taiwanese children.

**Methods:**

Taiwan's National Epidemiological Study of Child Mental Disorders used the stratified cluster sampling to select 69 schools in Taiwan resulting in a nationally representative sample of 4816 children in grades 3 (*n* = 1352), 5 (*n* = 1297) and 7 (*n* = 2167). All the participants underwent face-to-face psychiatric interviews using the Kiddie-Schedule for Affective Disorders and Schizophrenia-Epidemiological version, modified for the DSM-5, and they and their parents completed questionnaires. The inverse probability censoring weighting (IPCW)-adjusted prevalence was reported to minimise non-response bias.

**Results:**

The IPCW-adjusted prevalence rates of mental disorders decreased by 0.1–0.5% than raw weighted prevalence. The IPCW-adjusted weighted lifetime and 6-month prevalence rates for overall mental disorders were 31.6 and 25.0%, respectively. The most prevalent mental disorders (lifetime, 6-month) were anxiety disorders (15.2, 12.0%) and attention-deficit hyperactivity disorder (10.1, 8.7%), followed by sleep disorders, tic disorders, oppositional defiant disorder and autism spectrum disorder. The prevalence rates of new DSM-5 mental disorders, avoidant/restrictive food intake disorder and disruptive mood dysregulation disorder were low (<1%).

**Conclusions:**

Our findings, similar to the DSM-IV prevalence rates reported in Western countries, indicate that DSM-5 mental disorders are common in the Taiwanese child population and suggest the need for public awareness, early detection and prevention.

## Introduction

Although mental health issues across the lifespan have attracted tremendous attention in recent decades, mental illnesses in the child population remain a global public-health challenge (Patel *et al*., 2007). Because childhood-onset mental disorders are usually detected in later life, they are also very likely to cause functional impairment and psychopathology in adulthood (Costello *et al*., 2005). Hence, regular surveillance of mental conditions in the child population is the fundamental element to prevent mental illness and improve mental well-being across the lifespan.

Since 1977, the World Health Organization has suggested that every country should have child psychiatry plan and research (World Health Organization, 1977). However, only a few Western countries have conducted their national surveys, including Australia (Sawyer *et al*., 2001), Germany (Ravens-Sieberer *et al*., 2008), the Netherlands (Verhulst *et al*., 1997), Israel (Farbstein *et al*., 2010), Italy (Frigerio *et al*., 2009), the UK (Ford *et al*., 2003) and the USA (Merikangas *et al*., 2009; Kessler *et al*., 2012; Nock *et al*., 2013) (see online Supplementary Table S1). These mental disorders include attention-deficit hyperactivity disorder (ADHD; 2.2–11.2%), anxiety disorder (4.0–31.9%), major depressive disorder (MDD; 0.9–11.7%), oppositional defiant disorder (ODD; 1.8–12.6%) and conduct disorder (CD; 0.9–6.8%). Despite varied prevalence rates across these studies, they suggest that 25–30% of children and adolescents are affected by any mental disorder (Costello *et al*., 2005).

These epidemiological data from above mentioned national surveys may not be applied to Asian countries like Taiwan because of ethnic and cultural difference. It has been reported that the prevalence rates of mental disorders are relatively low in the Asian countries (Steel *et al*., 2014). However, an alternative hypothesis is that several common child mental disorders, such as ASD, tic disorders and ADHD, have strong biological and genetic bases. Thus, they should be relatively stable across countries (Robertson, 2008). Moreover, those national surveys were based on previous Diagnostic and Statistical Manual of Mental Disorders versions (DSM-III or DSM-IV) rather than the newly released DSM-5 since May 2013. There is a lack of empirical data on the prevalence and distribution of a wide range of mental disorders based on the DSM-5 from a representative sample of children. Some new disorders emerging in the DSM-5 occur during childhood, such as avoidant/restrictive food intake disorder (ARFID) and disruptive mood dysregulation disorder (DMDD) (American Psychiatric Association, 2013). The updated child psychiatric epidemiological data help clinicians to understand the impact of the change of diagnostic system and the current epidemic of new mental disorders in children.

Taiwan's National Epidemiological Study of Child Mental Disorders (TNESCMD) is the first national survey designed explicitly for child mental disorders in Taiwan. To assess a broad range of DSM-5 mental disorders, we used the Mandarin version of the Schedule for Affective Disorders and Schizophrenia for School-Age Children-Epidemiologic version (K-SADS-E) modified based on the DSM-5 (Chen *et al*., 2017). This paper reports the study methodology and lifetime and 6-month prevalence rates of DSM-5 mental disorders, which are compared with those reported in previous national surveys in Western countries.

## Method

The TNESCMD study was approved by the Research Ethics Committee of National Taiwan University Hospital, Taiwan (approval number: 201411056RINA), before study implementation.

### Sampling method

The TNESCMD is a school-based national epidemiological study of common mental disorders among children in Taiwan in 2015–2017. We collected the data via questionnaires (participants, parents and teachers) and psychiatric interviews (participants). The stratified (urbanicity levels and geographic strata) clustering sampling method was used to select a representative sample. There were 69 schools selected, and all classes in the selected grades (i.e. 3, 5, 7) of these schools were invited to participate in the study. Detailed information on sampling method and recruitment procedure are available in the online Supplementary Method.

### Sample

**Fig. 1.**
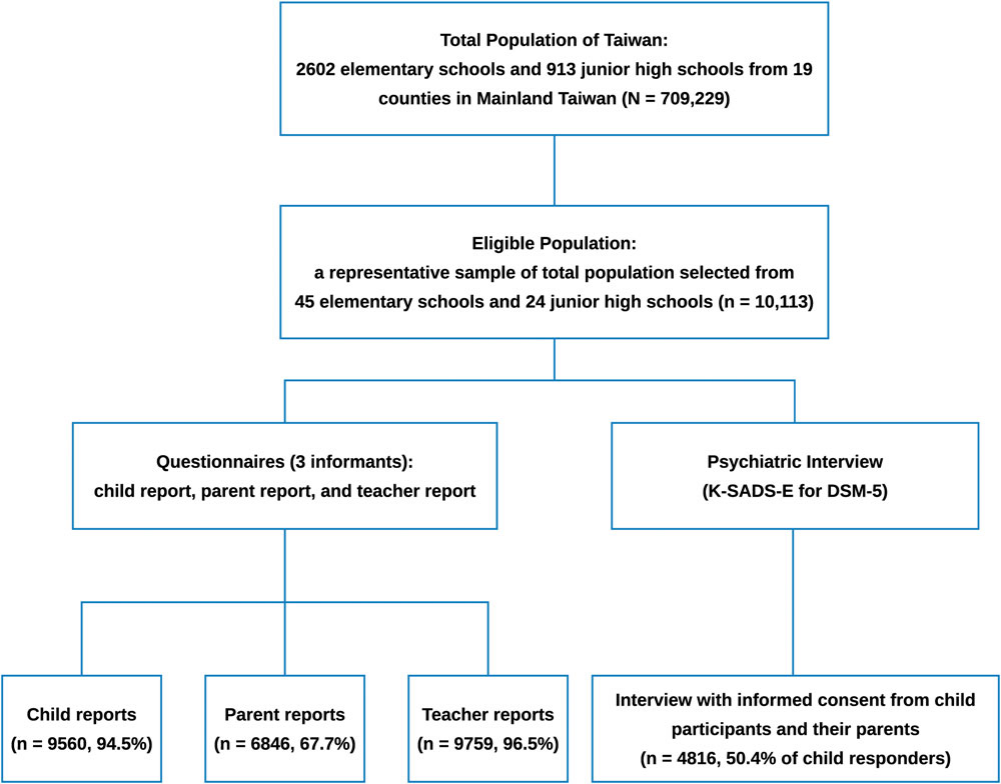
Sampling Procedure and Participant's Recruitment in the Taiwan's National Epidemiological Study of Child Mental Disorders.

Among the 10 118 eligible children selected via the stratified clustering sampling method, 9560 (94.4%) children, 6846 (67.6%) parents and 9759 (96.3%) teachers provided their written informed consent and completed the questionnaire-based survey from 1 June 2015 to 31 January 2017. Parents of the 9560 children also provide their written consent to agree on these children's participation in the questionnaires survey. For the psychiatric diagnostic interview, 4816 (47.6%; 2520 boys, 52.3%) children in grades 3 (*n* = 1352, 28.1; 693 boys, 51.2%), 5 (*n* = 1297, 26.9%; 666 boys, 51.4%) and 7 (*n* = 2167, 45.0%; 1161 boys, 53.6%) and their parents provided the written informed consent for the psychiatric interview with the participants. The response rate was 50.4% among 9560 children who completed the questionnaires (Figure 1). Half of the parents did not consent to their children's psychiatric interview at school mainly because they did not want their children to miss any class or to go home late due to the interview. Their age ranges were 7–10 (mean ± standard deviation, s.d. = 8.9 ± 0.4) years, 9–12 (mean ± s.d. = 10.9 ± 0.4) years and 12–14 (mean ± s.d. = 13.0 ± 0.4) years for grade 3, 5 and 7 students, respectively. Table 1 presents the demographic characteristics of the 4816 participants. The distributions of sex and urbanicity were not different from those of the target population (*p*-values > 0.05). Grade 7 children were more likely to participate in the survey, but after poststratification for adjusting the sampling weights, the grade distribution was equivalent to those of the entire population. With the weighted percentage, our sample was the representative of the Taiwanese population in 2013.

**Table 1.** Distribution of demographic features in the TNESCMD compared with that of the Taiwan student population in 2013

	TNESCMD			Taiwan student population in 2013^a^
Demographic variables	*N*	Unweighted (%)	Weighted (%)	*N* (%)
Sex				
Male	2520	52.3	51.8	370 076 (52.2)
Female	2296	47.7	48.2	339 153 (47.8)
Grade				
Third	1352	28.1	29.5	207 493 (29.3)
Fifth	1297	26.9	32.4	228 425 (32.2)
Seventh	2167	45.0	38.1	273 311 (38.5)
Urbanicity				
Urban	3413	70.9	70.1	493 533 (69.6)
Rural	1403	29.1	29.9	215 696 (30.4)

TNESCMD, Taiwan's National Epidemiological Study of Child Mental Disorders.

a Obtained from the Taiwan Ministry of Education conducted in 2013.

### Measurements

#### The Mandarin version of the K-SADS-E for DSM-5

The Chinese version of the K-SADS-E, a semi-structured clinical interview for the systematic assessment of mental disorders in children and adolescents in Taiwan (Gau *et al*., 2005), was further modified by Gau and colleagues based on the DSM-5 (Chen *et al*., 2017). The psychometric study of the Mandarin version of the K-SADS-E for DSM-5 revealed good inter-rater reliability (prevalence-adjusted, bias-adjusted *κ* = 0.80–1.00) and convergent and divergent validity (Chen *et al*., 2017). According to the DSM-5 criteria, we also assessed the functional impairment based on the participant's report of clinically significant distress or disturbance in several functional domains including social, family and school settings. We then incorporated functional impairment and symptoms criteria in the diagnosis of mental disorders, i.e. participants who had been given a diagnosis needed the presence of functional impairment if the diagnosis requires a criterion of functional impairment. Due to the feasibility, we only conducted the diagnostic interview with the child participants at school using the K-SADS-E in Mandarin. Moreover, questionnaires were used to obtain relevant information from their parents. In addition to the DSM-5 mental disorders, the K-SADS-E also included the suicide-related problems. Detailed information on interviewer training and suicide-related problems for the K-SADS-E in Mandarin and questionnaires is available in the online Supplementary Method.

#### Statistical analysis

Due to the lack of parental information in the diagnostic interview using the K-SADS-E, we examined the child–parent agreement on behavioural problems, Youth Self-Report and Parent Report Form of Child Behavior Checklist (YSR and CBCL, respectively), using the correlation analysis (Pearson's *r*). Cohen (1988) suggested the interpretation of effect size in Pearson's *r* with small for 0.1, moderate/medium for 0.3 and large for 0.5 (Cohen, 1988).

Two sample weighting methods were used in the TNESCMD. The unadjusted national population weight was used to estimate the prevalence due to unequal probabilities of selection and poststratification for adjusting the sampling weights. We also used the inverse probability censoring weighting (IPCW) method to minimise non-response bias, which indicates there may be differences in observed and unobserved variables between children who participated and those who did not. Before performing IPCW, we first conducted bias analyses to detect whether there was a non-response bias using relevant information obtained from parents’ questionnaires (see online Supplementary Method for detailed information about the bias analysis and IPCW).

We reported two different weighted prevalence rates, weighted prevalence with unadjusted national population weight and adjusted prevalence with IPCW and their 95% confidence interval (CI). The prevalence ratio (PR), the size of the difference, was used to present the comparison between unadjusted and IPCW-adjusted prevalence. The 95% CI was calculated using Katz's logarithm method. If the CI of the PR includes the null value of 1, there was no significant difference in the two prevalence rates.

## Results

### Child–parent agreement on child's behavioural problems

We found significantly low-to-moderate child–parent agreement on many subscales of the CBCL with a range of Pearson's *r* from 0.23 (the subscales of delinquent behaviour and thought problems) to 0.34 (the subscale of attention problems; online Supplementary Table S2).

### Non-response bias and IPCW-adjusted prevalence

According to the bias analysis, we found that parents who provided written informed consent for their children to receive the psychiatric interviews were younger, had more severe depression and anxiety symptoms based on the Adult Self-(Report Inventory-Anxiety and Depression score; Yeh *et al*., 2008), reported poorer self-perceived health (based on the Chinese Health Questionnaire score; Cheng and Williams, 1986), lower perceived family function (based on the Family Adaptation, Partnership, Growth, Affection, and Resolve score; Gau *et al*., 2009), their children had more behavioral problems (based on the CBCL; Shang *et al*., 2006) and more behavioral problems at home (based on the subscale of home behaviors on the Social Adjustment Inventory for Children and Adolescents; Gau *et al*., 2006) than their counterparts (online Supplementary Table S3).

The results suggested a possible non-response bias; hence, we conducted IPCW to adjust for the weighted prevalence rates. After adjustment for the possible non-response bias, we found that IPCW-adjusted prevalence rates of mental disorders decreased by 0.1–0.5% (online Supplementary Table S4), but there were no significant differences in any of the comparison between the unadjusted weighted prevalence and IPCW prevalence rates (all the 95% CIs of the PR included 1, see online Supplementary Table S4).

### Lifetime and 6-month prevalence of mental disorders

Table 2 presents the IPCW-adjusted lifetime and 6-month weighted prevalence rates with their 95% CIs of mental disorders according to the DSM-5 diagnostic criteria in all the respondents among the third-, fifth- and seventh-grade children. Prevalent disorders (>1%) included anxiety disorders (15.2 and 12.0%), sleep disorders (12.0 and 6.2%), ADHD (10.1 and 8.7%), tic disorders (2.6 and 2.1%), ODD (2.0 and 1.5%), MDD (1.7 and 0.7%), obsessive–compulsive disorder (1.4 and 0.8%) and autism spectrum disorder (ASD, 1.0%; only lifetime prevalence). Other mental disorders, such as CD, intermittent explosive disorder, persistent depressive disorders, feeding and eating disorders, post-traumatic stress disorder and gender dysphoria, were <1% (Table 2). Only one child had lifetime bipolar I disorder. Overall, the weighted lifetime prevalence and 6-month prevalence of at least one kind of mental disorder were 31.6 and 25.0%; and at least two kinds of mental disorders were 12.9 and 8.3%, respectively (Table 2). A total of 40.8 and 24.6% of all affected children with lifetime and 6-month mental disorder also suffered from the additional mental disorder, respectively. For suicide-related problems, the lifetime and 6-month weighted prevalence rates were 8.2 *v*. 3.1% for suicidal ideation, 3.6 *v*. 1.7% for suicidal plan and 0.7 *v*. 0.3% for suicidal attempts.

**Table 2.** The adjusted weighted and 6-month weight prevalence of diagnostic distribution of DSM-5 mental disorders in Taiwan's National Epidemiological Study of Child Mental Disorders

	IPCW-adjusted prevalence					
	Lifetime			six-month		
DSM-5 diagnoses	*N*	_wt_%	95% CI	*N*	_wt_%	95% CI
Neurodevelopmental disorders						
Autism spectrum disorder^a^	52	1.0	0.6–1.5	–	–	–
ADHD	487	10.1	8.9–11.3	412	8.7	7.3–10.1
Tic disorders	151	2.6	2.0–3.4	126	2.1	1.4–2.7
DICCD						
Oppositional defiant disorder	97	2.0	1.4–2.6	76	1.5	0.9–2.1
Conduct disorder	15	0.5	0.1–0.9	9	0.1	0.0–0.3
Intermittent explosive disorder	17	0.2	0.0–0.4	10	0.1	0.0–0.3
Depressive disorders						
Major depressive disorder	79	1.7	0.7–2.7	24	0.7	0.0–1.5
Persistent depressive disorder	28	0.8	0.0–1.6	15	0.2	0.0–0.4
DMDD	14	0.3	0.1–0.5	12	0.2	0.0–0.4
Anxiety disorders						
Any anxiety disorder	702	15.2	12.7–17.7	550	12.0	10.0–14.0
Generalised anxiety disorder	33	0.9	0.3–1.5	30	0.7	0.3–1.1
Social anxiety disorder	154	3.6	2.6–4.6	137	2.7	1.9–3.5
Specific phobia disorder	412	8.7	6.9–10.5	366	7.7	6.1–9.3
Separation anxiety disorder	178	4.4	3.2–5.6	59	2.2	0.8–3.6
Panic disorder	19	0.4	0.0–0.8	8	0.1	0.0–0.2
Agoraphobia	13	0.4	0.0–0.8	13	0.4	0.0–0.8
Feeding and eating disorders						
Anorexia nervosa	4	0.2	0.0–0.4	3	0.2	0.0–0.4
ARFID	40	0.5	0.3–0.7	22	0.3	0.1–0.5
Obsessive–compulsive disorder	47	1.4	0.2–2.6	26	0.8	0.0–1.6
Schizophrenia	4	0.1	0.0–0.3	4	0.1	0.0–0.3
Gender dysphoria	14	0.3	0.1–0.5	13	0.3	0.1–0.5
Reactive attachment disorder	4	0.1	0.0–0.2	4	0.1	0.0–0.2
Post-traumatic stress disorder	7	0.1	0.0–0.2	0	0.0	0.0–0.0
Dissociative identity disorder	3	0.4	0.0–0.8	1	0.2	0.0–0.4
Any sleep disorders	473	12.0	9.1–14.9	274	6.2	4.8–7.6
Insomnia disorder	105	2.2	1.3–3.0	92	1.8	0.9–2.7
Hypersomnolence disorder	3	0.1	0.0–0.3	3	0.1	0.0–0.3
Circadian rhythm sleep–wake disorders	2	0.1	0.0–0.2	2	0.1	0.0–0.2
Nightmare disorder	337	8.9	6.3–11.4	171	4.1	2.9–5.3
NREM sleep arousal disorders – sleepwalking	63	1.4	1.0–1.8	23	0.4	0.2–0.6
NREM sleep arousal disorders – sleep terror	27	0.4	0.2–0.6	13	0.1	0.0–0.2
Restless legs syndrome	8	0.3	0.0–0.7	8	0.3	0.0–0.7
Total disorders, No.^b^						
Any	1499	31.6	28.1–35.3	1187	25.0	22.1–27.9
⩾2	611	12.9	10.5–14.2	416	8.3	6.8–9.9
⩾3	244	5.6	4.1–7.0	149	3.1	2.3–3.8
Suicide-related problems						
Suicidal ideation	377	8.2	6.0–10.4	163	3.1	2.3–3.9
Suicidal plan	153	3.6	2.2–5.0	77	1.7	0.7–2.7
Suicidal attempt	43	0.7	0.3–1.1	19	0.3	0.1–0.5

ADHD, attention-deficit hyperactivity disorder; ARFID, avoidant/restrictive food intake disorder; DMDD, disruptive mood dysregulation disorder; DMDD, disruptive mood dysregulation disorder; DICCD, disruptive, impulse-control and conduct disorders; NREM, non-rapid eye movement; IPCW, inverse probability censoring weighting.

a Autism spectrum disorder is considered a lifelong disorder; only lifetime weighted prevalence was reported.

b Total disorders do not include suicide-related problems.

## Discussion

To the best of our knowledge, the current study is the first to provide a very comprehensive assessment of a wide range of DSM-5 disorders based on a semi-structured psychiatric interview (K-SADS-E) among few national prevalence surveys of mental disorders in youth populations (Verhulst *et al*., 1997; Sawyer *et al*., 2001; Ford *et al*., 2003; Ravens-Sieberer *et al*., 2008; Frigerio *et al*., 2009; Merikangas *et al*., 2009; Farbstein *et al*., 2010). Our main findings are the IPCW-adjusted weighted lifetime and 6-month prevalence rates for overall mental disorders, which were 32.2 and 25.8%, respectively, with the most prevalent mental disorders as anxiety disorders (15.2, 12.0%) and ADHD (10.1, 8.7%), followed by sleep disorders, tic disorders, ODD and ASD.

### Prevalence of mental disorders

While reading our comparison of the results from the current study with those from the national surveys in other countries, it should be noted that there were differences in methodologies, e.g. the respondents’ age range, measurements, diagnostic criteria, the time frame of the prevalence estimate and sampling methods. These methodological differences may explain to a considerable extent for the differential prevalence rates of child mental disorders across studies.

The lifetime prevalence rate of total mental disorders in children estimated in this study (31.6%) was within the range of figures reported from national studies in the USA [lifetime: 49.5% (Merikangas *et al*., 2010) and 12-month: 40.3% (Kessler *et al*., 2012) for subjects 13–18 years old] and the Netherlands [6-month: 35.5% (Verhulst *et al*., 1997) for subjects 4–18 years old] using diagnostic interviews, but was considerably higher than national surveys in other countries [current: 9.5–19.2% (Sawyer *et al*., 2001; Ford *et al*., 2003; Ravens-Sieberer *et al*., 2008; Frigerio *et al*., 2009; Farbstein *et al*., 2010) for subjects 4–17 years old] that mainly employed the development and well-being assessment to assess youth's psychopathology. Furthermore, regarding comorbidity in the TNESCMD, our finding that 40.8 and 24.6% affected children would report other additional lifetime and 6-month mental disorder is highly close to the USA (lifetime: 40%) (Merikangas *et al*., 2010). Overall, we found that the prevalence rates of total mental disorders in children were similar across the East and West, despite different DSM versions used. One possible explanation might be that the prevalence rates of several common child mental disorders are more likely to be affected by the assessment tools rather than the diagnostic system.

### ADHD and MDD

Changes in the diagnostic criteria from the DSM-IV to the DSM-5 may proportionally account for the differences in the prevalence rates of certain mental disorders between this study and other national surveys. For example, the lifetime and 6-month prevalence rates of ADHD in this study (10.1 and 8.7%, respectively) were largely similar to those from the Australian national survey (12-month prevalence: 11.2%) (Sawyer *et al*., 2001), but were higher than figures from other national surveys (2.2–8.7%) (Verhulst *et al*., 1997; Ford *et al*., 2003; Ravens-Sieberer *et al*., 2008; Frigerio *et al*., 2009; Merikangas *et al*., 2009; Farbstein *et al*., 2010; Kessler *et al*., 2012). The increase in ADHD prevalence might be attributable to the fact that the DSM-5 has widened the threshold for ADHD diagnosis (van de Glind *et al*., 2014). For example, the onset age of ADHD changed from ‘before the age of 7 years’ in the DSM-IV to ‘before the age of 12 years’ in the DSM-5, the change of descriptions for the criteria of ADHD as ‘*clinically significant impairment*’ to ‘*interfere with or reduce the quality*’, and the addition of ‘*taps hands*’ in the first symptom of hyperactivity–impulsivity in the DSM-5.

Similarly, for MDD, the minimum duration of MDD required was shortened from ‘4 *weeks*’ in the DSM-IV to ‘2 *weeks*’ in the DSM-5. This change may at least in part account for the increased 6-month prevalence rate of MDD in the present study compared with previous Taiwan's research (Gau *et al*., 2005). The increase in the prevalence of these two disorders from the DSM-IV to the DSM-5 has also been reported in other studies (Uher *et al*., 2014; van de Glind *et al*., 2014).

### ODD/CD

Although the lifetime (2.0%) and 6-month (1.5%) prevalence rates of ODD in this study were significantly lower than figures in the US national survey (lifetime: 12.6% and 12-month: 8.3%) (Merikangas *et al*., 2009; Kessler *et al*., 2012), they were similar to those in national surveys from the UK (2.3%) (Ford *et al*., 2003) and Israel (1.8%) (Farbstein *et al*., 2010). On the other hand, our prevalence rates of CD (lifetime: 0.5% and 6-month: 0.1%) were significantly lower than the rates from all other national surveys (range from 0.9 to 9.7%) (Verhulst *et al*., 1997; Sawyer *et al*., 2001; Ford *et al*., 2003; Ravens-Sieberer *et al*., 2008; Frigerio *et al*., 2009; Merikangas *et al*., 2009; Kessler *et al*., 2012; Farbstein *et al*., 2010). One possible explanation is that ODD was usually identified in late childhood or early adolescence, whereas CD usually occurs during adolescence (Rowe *et al*., 2010). Our participants were children aged 7–15 (grades 3, 5 and 7), much younger than their counterparts in other national surveys (13–18 years old). Moreover, Asian youths were less likely to have CD compared with their Western counterparts at similar ages (Sakai *et al*., 2008).

### Anxiety disorders

This study reported that anxiety disorders, including GAD, social anxiety disorder, specific phobia disorder, SAD, panic disorder and agoraphobia, were the most common mental disorders (lifetime: 15.2% and 6-month: 12.0%), with the rates of specific phobia disorder as the highest and agoraphobia and panic disorder as the lowest. These figures of anxiety disorders were similar to those from the majority of national surveys (Verhulst *et al*., 1997; Farbstein *et al*., 2010; Merikangas *et al*., 2010; Kessler *et al*., 2012). The comparable rates between the two DSM versions suggest that patterns of anxiety disorders occurring in the child population are similar between the DSM-IV and the DSM-5 criteria. Given their high prevalence, anxiety disorders in children may greatly affect the child, his/her family and parent–child interactions (Ginsburg *et al*., 2004). Furthermore, although the extent of disability and relevant impairment resulting from anxiety disorders was lower than major mental disorders (Baxter *et al*., 2014), the development of anxiety disorders in young children and adolescents was reported to be associated with increased risks of developing sequential depression and functional impairment in adulthood (Bongers *et al*., 2003). As anxiety disorders in children have received relatively less attention from clinicians, family, educators and society, our findings strongly suggest the need for primary prevention, early detection and intervention.

### ASD

The prevalence of ASD was 1.0% in this study, higher than the results from several earlier reports and the rate from a global meta-analysis (0.62%) (Elsabbagh *et al*., 2012). However, this figure was close to the recent estimate of ASD prevalence up to 2.2% in the National Health Interview Survey by the Centers for Disease Control and Prevention in the USA (Zablotsky *et al*., 2015) and 2.6% in South Korea (Kim *et al*., 2011). Although the elevated rates in recent surveys might have been derived from the differences in research methodology, they might also be attributed to an increased awareness of ASD in the past few decades. This highlights the importance of long-term care needs for individuals with ASD and their families in Taiwan and around the globe.

### Suicide-related problems

The current study presents the first national data on the lifetime and 6-month prevalence rates of suicide-related problems among children in Taiwan: suicidal ideation (8.2 *v*. 3.1%), suicidal plans (3.6 *v*. 1.7%) and suicidal attempts (0.7 *v*. 0.3%). Comparatively, Germany (current: 3.8 *v*. 2.9%) (Ravens-Sieberer *et al*., 2008) and the USA (lifetime: 12.1 *v*. 4.1%) (Nock *et al*., 2013) had a similar prevalence of suicide ideation but a higher prevalence of suicidal attempts. These differences in suicide attempts are possibly due to the age difference between the study populations, as suicidal attempts are more likely to be observed in adolescents and young adults than children, and our participants were younger than those in the national surveys of Germany and the USA (Tang *et al*., 2009). Combing our results and the report of suicide as the second-leading cause of death in the Taiwanese adolescent and early adult populations (Ministry of Welfare and Health, 2017), the efforts of suicide prevention should be prioritised in the mental well-being plans for the young population. Several relevant issues warrant further investigation: (1) understanding the transition and risk factors from suicidal ideation to suicidal attempts, then to actual suicide in children; (2) comorbidities between suicide-related problems and mental disorders in children, especially for impulsivity, hyperactivity and depressive disorders; and (3) the family as an essential resource for the early detection of a suicide risk and the prevention of related injuries and death in children.

### Sleep disorders

This study is one of the first to comprehensively examine the prevalence of sleep disorders diagnosed according to the DSM-5 criteria among all national surveys. Our findings indicated that the prevalence rates of any sleep problems and sleep disorders were common in children (lifetime: 12.0% and 6-month: 6.2%). Among all types of sleep disorders examined in this study, nightmare disorder was the most common, followed by insomnia disorder and non-rapid eye movement (NREM) sleep arousal disorders – sleepwalking. The other sleep disorders were comparatively uncommon, with prevalence lower than 1%, including NREM sleep arousal disorders – sleep terror, hypersomnolence, circadian rhythm sleep–wake disorders, restless legs syndrome and narcolepsy. One possible reason for the high prevalence of nightmare disorder may be that it has no minimum duration requirement in the diagnostic criteria, unlike most of the other mental disorders included in the DSM-5. On the other hand, the low prevalence rates of some sleep disorders, such as hypersomnolence and narcolepsy, which are more prevalent in adolescents than children (Millman, 2005), may partially result from the lack of objective measures during our field survey, including polysomnography or actigraphy (Kushida *et al*., 2001). Sleep disorders in children have significant impacts on their cognitive development, school function and distress for families. Hence, effective prevention and intervention of sleep disorders in children are warranted to improve their sleep quality and to exert a positive effect on their psychological well-being.

### Different prevalence rates from treated populations

In contrast to the low prevalence rates of treated cases of child mental disorders such as ADHD and ASD in Taiwan, our findings revealed that DSM-5 mental disorders were highly prevalent among Taiwanese children. For example, the treated prevalence rates of ASD and ADHD were reported to be 0.28% (Chien *et al*., 2011) and 1.64% (Chien *et al*., 2012), respectively, based on a dataset from the National Health Insurance in Taiwan. Such discrepancy suggests a low level of awareness about mental disorders and inadequate mental health service in the childhood population. There is room for improvement in the assessment, treatment and prevention of child mental disorders.

### Limitation

Some major limitations should be mentioned. First, although our study is a national survey, some rare mental disorders in children, such as bipolar II or cyclothymic disorders, were not found in this age-restricted sample. Second, the diagnostic interview using the K-SADS-E should optimally be conducted with both children and their parents. In this school-based survey, we solely interviewed the children because it was not feasible to ask the parents to participate in the interview at the school. However, we explored the child–parent agreement and found moderate child–parent agreement on many children's behavioural problems (based on the CBCL score; online Supplementary Table S2) and previous study also demonstrated that a moderate to high child–parent agreement was found in the diagnostic interview across a variety of child mental disorders (Hodges *et al*., 1990). Nevertheless, further studies should still be cautious with these assessment limitations. Third, this survey adopted voluntary recruitment and was non-mandatory, depending on the cooperation and willingness of the school teachers, child participants and their parents. A 50.4% response rate for diagnostic interview in our study suggests that our results might suffer from a certain degree of non-response bias. Nevertheless, we conducted a bias analysis to address this potential bias and used IPCW to minimise such bias. Regarding non-response bias, we found that the children who underwent the clinical interview (with informed consent provided) had more symptoms of a wide range of emotional and behavioural problems compared with the children who did not receive the interview due to a lack of informed consent from their parents (Table 2). This finding was contradictory to those of earlier studies in other countries showing that children without the parental consent of interview might have more severe psychopathologies with social withdrawal and aggression (Frame and Strauss, 1987; de Winter *et al*., 2005). A possible reason for the differential finding may be the increased awareness of child mental disorders. Furthermore, the non-response bias might have little impact on our results of the prevalence rates of mental disorders because the prevalence rates before and after the adjustment of IPCW did not differ significantly, suggesting that our results provided robust estimates of the national prevalence of mental disorders in children. Nevertheless, further studies should still be cautious with the possible non-response bias while interpreting our results.

## Conclusions

The first national epidemiological study of DSM-5 child mental disorders has provided substantial evidence to indicate that mental disorders in Taiwanese children are common and their rates corresponded to the DSM-IV prevalence rates reported in Western countries. The increasing trends of ADHD, MDD and ASD may be explained by the change of diagnostic criteria in the DSM-5 or increasing awareness of child mental disorders. Emerging evidence supports the existence of some new DSM-5 diagnoses such as ARFID and DMDD. Our study indicates the need for regular surveys of mental disorders in children to assess their changing trends. Findings from such surveys can increase public awareness of child mental disorders and highlight the priorities for future mental health policies including prevention, early detection and intervention for youth population.

## Supplementary material

For supplementary material accompanying this paper visit http://dx.doi.org/10.1017/S2045796018000793. click here to view supplementary material

## Availability of data and materials

These data were owned by the Taiwan Ministry of Health and Welfare. If researchers are interested in applying the data from Taiwan Ministry of Health and Welfare, information is available at http://www.mohw.gov.tw.
