# Supplementary material for: Prevalence of DSM-5 mental disorders in a nationally representative sample of children in Taiwan: methodology and main findings
Source: Epidemiol Psychiatr Sci. 2019 Jan 30;29:e15. doi: 10.1017/S2045796018000793 (PMC8061245; doi:10.1017/S2045796018000793)
Supplement: Supplementary file 1 [file S2045796018000793sup001.docx]

Supplementary material (Online-only)

**List:**

**Supplementary Method.**

**Supplementary Table S1.** Summary of national surveys of mental disorders in children from different countries

**Supplementary Table S2.** Child-parent agreement on behavioral problems in children in the sample of Taiwan’s National Epidemiological Study of Child Mental Disorders

**Supplementary Table S3.** The socio-demographic features, psychological, and clinical traits between children with and without K-SADS-E interview in the sample of Taiwan’s National Epidemiological Study of Child Mental Disorders

# Supplementary Table S4. The weighted and 6-month weight prevalence of diagnostic distribution of DSM-5 mental disorders in Taiwan's National Epidemiological Study of Child Mental Disorders

# Supplementary Method

### *Sampling method*

The school sampling frame was constructed using the school lists in 2013 from the Ministry of Education, Taiwan. Nineteen counties in Taiwan were stratified into seven major geographic regions: highly urbanized cities, moderately urbanized cities, boomtowns, general cities, aging cities, agriculture cities, and remote areas. The categorization of the seven regions was based on five variables: (1) the population density, (2) the population ratio of people with educational levels of college or above, (3) the population ratio of people over the age of 65, (4) the population ratio of agricultural workers, and (5) the number of physicians per 100,000 people. The region of highly urbanized cities represents the highest and that of the remote areas the least urbanicity (Liu *et al.*, 2006). Due to the analysis of the standard errors of the prevalence of mental disorders and budget of financial cost for each sampled unit, we decided to recruit 4500-5000 children and 9000-10000 children and their parents and teachers for diagnostic psychiatric interview and questionnaires, respectively, within a limited study period, i.e., two years.

## Procedure

The sampled schools received official documents and information from the Ministry of Education to participate in the TNESCMD. We then contacted these schools to clearly explain the purpose and procedure of this study and reassurance of the confidentiality of the data and voluntary participation of each student and family. We then scheduled recruitment and assessment procedures. The children and their parents and teachers received the informed consent in paper format with a complete description of the purpose and procedure of this study with the reassurance of confidentiality and having an opportunity to ask questions. They submitted their written informed consent with a reserved, sealed envelope to their teachers. Teachers would collect all participating children and their parents' and their own informed consent to the questionnaire survey, and then forward to research assistants for the administration of questionnaires to those providing written informed consent. The participants received an honorarium of NT$200 (approximately US$7) after completing the questionnaire survey and the interview at the school.

### *Suicide-related problems*

In addition to the DSM-5 mental disorders, the K-SADS-E also included the suicide-related problems in its DSM-IV and current DSM-5 versions. We also screened three suicide-related problems in the TNESCMD, i.e., suicidal ideation, plan, and attempt. Suicidal ideation refers to the thoughts to end one’s own life. Suicide plan refers to the formulation of proposed methods to end one’s life given that the index person has suicidal ideation. Suicidal attempt refers to the engagement in potentially self-injurious behavior with a deadly idea of ending one’s own life.

### *Interviewer training and fieldwork*

Eight interviewers participated in this survey. They earned a bachelor or master degree in psychology with extensive experience working with children and families, and had received intensive clinical training for sufficient knowledge of child mental disorders, and clinical and psychiatric interviewing skills with children. The training program, lasting at least six months, included reading, observation, practice, case presentation and discussion, and structured and semi-structured interviews using the K-SADS-E. Subsequently, each interviewer interviewed ten clinical subjects independently, followed by the confirmation and discussion with the corresponding author and trainers. They were qualified as the interviewers for this study only if the ratings of theirs were at least 90% matched with the corresponding author. During the study period, all the interviews (part of them were recorded for quality control and diagnosis discussion if the students and their parents consented) and data collection were reviewed and discussed on a weekly basis.

### *Questionnaires*

For further obtaining information from participating children's parents, we examined their emotional symptoms (Adult Self-Report Inventory, ASRI–Anxiety and Depression) (Yeh *et al.*, 2008), self-perceived health (Chinese Health Questionnaire, CHQ) (Cheng and Williams, 1986), and parent's report of family function (Family Adaptation, Partnership, Growth, Affection, and Resolve, Family APGAR) (Gau *et al.*, 2009), behavioral problems (Child Behavior Checklist, CBCL) (Shang *et al.*, 2006), and social school function (Social Adjustment Inventory for Children and Adolescents, SAICA) (Gau *et al.*, 2006).

### *Bias analysis*

The χ2 for categorical variables and independent *t*-test for continuous variables were used to explore whether their parents' sociodemographics (parental average age and education level), emotional symptoms (ASRI–Anxiety and Depression), self-perceived health (CHQ), and parent's report of family function Family APGAR), behavioral problems (CBCL), and social school function (Social Adjustment Inventory for Children and Adolescents, SAICA) differed between children with and without parental informed consent for participation of K-SADS-E interview. A correction of the *p*-value for multiple tests was conducted using the false discovery rate method.

*Inverse probability censoring weighting* (IPCW)

For IPCW, the logistic regression analysis with the participation status of the K-SADS-E interview as an outcome and those significant variables in the bias analysis as auxiliary information were used to obtain the censoring probability. The IPCW weight was obtained according to the reweighted prevalence rate using a combination of the weight of censoring probability and the unadjusted national population weight.

# Supplementary Table S1. Summary of national surveys of mental disorders in children from different countries.

| Countries | Netherlands^a^ | Australia | United Kingdom | United States | | Germany | Israel | Italy |
| --- | --- | --- | --- | --- | --- | --- | --- | --- |
| First author | Verhulst | Sawyer | Ford | Merikangas | Kessler | Rravens-  Sieberer | Farbstein | Frigerio |
| Measure of prevalence | 6-month^a^ | 12-month | Current prevalence: time frame of disorders changed according to their diagnostic criteria | Lifetime | 12-month | Current prevalence: time frame of disorders changed according to corresponding applied questionnaires | Current prevalence: time frame of disorders changed according to their diagnostic criteria | Current prevalence: time frame of disorders changed according to their diagnostic criteria |
| Study period | 1993 | 1997-1998 | 1999 | 2001-2003 | | 2003-2006 | 2004-2005 | unspecified |
| Publication Year | 1997 | 2001 | 2003 | 2010 | 2012 | 2008 | 2010 | 2009 |
| Assessment | CBCL: Youth Self Report & Teacher's Report Form and DISC for Parent version and child version (DSM-III) | CBCL: Youth Self Report & Teacher's Report Form and SCID (DSM-IV) | DWBA | A modification of the Composite International Diagnostic Interview | | Screen for  Child Anxiety Related Emotional Disorders for anxiety disorder; Center for Epidemiological Studies Depression Scale  for Children for depression; Child Behavior Checklist for conduct disorder; German ADHD Rating scale and Conner's’ Scale for ADHD | DWBA | CBCL: Youth Self Report & Teacher's Report Form and  DWBA |
| Sampling method | Multi-stage cluster sampling | Multi-stage sampling | Stratified multi-stage sampling | Stratified multi-stage sampling | | Stratified two-stage sampling | Two-stage cluster sampling | Two-stage cluster sampling |
| Sample size | 2916 | 4509 | 10,438 | 9244 | | 17641 | 957 | 3418 |
| Participant's age range | 4-18 | 4-17 | 5-15 | 13-18 | | 7-17 | 14-17 | 10-14 |
| Examined risk factors | demographics, comorbidities, and social class | demographics, quality of life, risk behaviors, and services of mental health | demographics, services of mental health, family function, school performance, stress emulation, social adjustment, and physical health | demographics, comorbidities, social class, sexual behaviors, environmental- and stress-related factors, family-related factors,  peer relationships, saliva | | demographics, rural-urban factor, family and parenting style, and social class | demographics, social class, learning disability status, physical health, chronic diseases, accident, exercise, and social support | demographics, comorbidities, social class, social factors, family factors, school-related factors, emotional difficulties, and genetic factors |
| ADHD | 2.6 | 11.2 | 2.2 | 8.7 | 6.5 | 3.9 (aged 7-10)  2.2 (aged 11-17) | 3.0 | - |
| ODD | - | - | 2.3 | 12.6 | 8.3 | - | 1.8 | - |
| Conduct disorder | 6.0 | 3.0 | 1.4 | 6.8 | 5.4 | 8.7 (aged 7-10)  9.7 (aged 11-17) | 0.9 | - |
| ^b^Major Depressive disorder | 3.6 | 3.0 | 0.9 | 11.7 | 8.2 | 5.6 (aged 7-10)  4.9 (aged 11-17) | 3.3 | 3.8 |
| Bipolar disorder | - | - | - | 2.9 | 2.1 | - | - | - |
| Anxiety disorder | 23.5 |  |  | 31.9 | 24.9 | 6.3 (aged 7-10)  4.0 (aged 11-17) |  |  |
| Generalized anxiety disorder | 1.3 | - | 3.8 | 2.2 | 1.1 | - | 6.1 | 6.5 |
| Specific phobia disorder | 12.7 | - | 1.0 | 19.3 | 15.8 | - | 2.8 | 0.8 |
| Social anxiety disorder | 9.2 | - | 0.3 | 9.1 | 8.2 | - | 0.9 | 0.4 |
| Panic disorder | 0.4 | - | 0.1 | 2.3 | 1.9 | - | 0.4 | 0.2 |
| Agoraphobia | 2.6 | - | 0.1 | 2.4 | 1.8 | - | - | 0.3 |
| Separation anxiety disorder | - | - | - | 7.6 | 1.6 | - | - | - |
| OCD | 1.0 | - | 0.3 | - | - | - | 1.2 | - |
| Bulimia nervosa | 0.3 | - | 0.1 (combined with eating disorders) | 2.7 (combined with eating disorders) | 2.8 (combined with eating disorders) | - | - | - |
| Anorexia nervosa | 0.3 | - |  |  |  | - | - | - |
| Tourette Syndrome | 0.1 | - | 0.1 | - | - | - | - | - |
| Substance use disorder | 3.5 | - | - | 11.4 | 8.3 | 1.6% for alcohol use disorder; 1.2% for drug use disorder (aged 11-17) | - | - |
| PTSD | - | - | 0.1 | 5.0 | 3.9 | - | 0.8 | - |
| Any mental disorder | 35.5 | - | 9.5 | 49.5 | 40.3 | - | 11.7 | 8.2 for SCID; 9.8 for CBCL |
| Suicide-related problems |  |  |  |  |  |  |  |  |
| Ideation | - | - | - | 12.1^b^ | - | 3.8 (aged 11-17) | - | - |
| Plan |  |  |  | 4.0^b^ |  |  |  |  |
| Behavior/Attempt | - | - | - | 4.1^b^ | - | 2.9 (aged 11-17) | - | - |

ADHD: Attention-deficit hyperactivity disorder; CBCL = Child Behavior Checklist; DISC = Diagnostic Interview Schedule for Children; DWBA = Development and Well-Being Assessment; OCD = Obsessive-compulsive disorder; ODD = Oppositional defiant disorders; SCI = The Structured Clinical Interview for The Diagnostic and Statistical Manual of Mental Disorders; PTSD = Post-traumatic stress disorder

^a^The prevalence of mental disorders of the national survey in the Netherlands was based on the Parent or Child versions of the Diagnostic Interview Schedule for Children. The results of the prevalence rates of suicide-related problems in the United States were extracted from Nock et al., 2013.

**Supplementary Table S2. Child-parent agreement on behavioral problems in children** **in the sample of Taiwan’s National Epidemiological Study of Child Mental Disorders**

|  | Youth Self-Report | Parent Report Form |  |
| --- | --- | --- | --- |
| Child Behavior Checklist | N=4240 | N=4240 | Correlation (*r*) |
| Aggressive behavior | 4.08 (5.44) | 4.6 (5.03) | 0.29 |
| Anxious/depressed | 3.57 (4.65) | 3.13 (3.82) | 0.27 |
| Attention problems | 3.79 (3.81) | 3.6 (3.48) | 0.34 |
| Delinquent behavior | 1.98 (3.17) | 1.43 (2.16) | 0.23 |
| Social problems | 2.20 (2.48) | 2.1 (2.25) | 0.33 |
| Somatic complaints | 1.90 (2.98) | 1.17 (2.10) | 0.25 |
| Thought problems | 1.80 (2.38) | 1.13 (1.62) | 0.23 |
| Withdrawn | 2.71 (2.94) | 2.18 (2.47) | 0.28 |
| Internalizing problems | 8.20 (9.44) | 6.51 (7.40) | 0.28 |
| Externalizing problems | 6.07 (8.27) | 6.05 (6.95) | 0.28 |

Note: Among 4816 children who were allowed to undergo the clinical interview, only 4240 parents and their children completed the Parent Report Form and Youth Self-Report of Child Behavior Checklist.

**Supplementary Table S3. The socio-demographic features, psychological, and clinical traits between children with and without K-SADS-E interview in the sample of Taiwan’s National Epidemiological Study of Child Mental Disorders**

|  | Children | |  | Statistics | | | | | | | | | |
| --- | --- | --- | --- | --- | --- | --- | --- | --- | --- | --- | --- | --- | --- |
| Variables | Without  K-SADS-E interview | With  K-SADS-E interview |  |  |  |  |  |  |  |  |  |  |  |
|  | N=2606 | N=4240 |  | χ2 or *t* | | Raw p | | | | Adjusted p | | | |
|  | N (%) | N (%) |  |  |  |  |  |  |  |  |  |  |  |
| Father's education level |  |  |  |  | |  | | | |  | | | |
| Elementary school | 31 (1.32) | 43 (1.09) |  | 2.0 | | 0.734 | | | | 0.777 | | | |
| Junior high school | 254 (10.84) | 435 (11.07) |  |  |  |  |  |  |  |  |  |  |  |
| Senior high school | 1165 (49.72) | 1909 (48.6) |  |  |  |  |  |  |  |  |  |  |  |
| College | 615 (26.25) | 1073 (27.32) |  |  |  |  |  |  |  |  |  |  |  |
| Graduate school | 277 (11.82) | 468 (11.91) |  |  |  |  |  |  |  |  |  |  |  |
| Mother's education level |  |  |  |  | |  | | | |  | | | |
| Elementary school | 51 (2.16) | 98 (2.49) |  | 3.8 | | 0.436 | | | | 0.523 | | | |
| Junior high school | 216 (9.16) | 344 (8.74) |  |  |  |  |  |  |  |  |  |  |  |
| Senior high school | 1303 (55.24) | 2105 (53.45) |  |  |  |  |  |  |  |  |  |  |  |
| College | 642 (27.21) | 1148 (29.15) |  |  |  |  |  |  |  |  |  |  |  |
| Graduate school | 147 (6.23) | 243 (6.17) |  |  |  |  |  |  |  |  |  |  |  |
|  | Mean (SD) | Mean (SD) |  |  | |  | | | |  | | | |
| Parental average age | 42.6 (5.16) | 42.2 (4.95) |  | 1.1 | | 0.025 | | | | 0.032 | | | |
| Family function  (Family APGAR) | 7.62 (2.73) | 7.38 (2.77) |  | 3.4 | | 0.001 | | | | 0.002 | | | |
| Emotional symptoms (Adult Self-Report Inventory-Anxiety and Depression) | | | | | | | | |  | | | |  |
| Anxiety | 5.19 (3.95) | 5.79 (4.12) |  | 5.6 | | <.001 | | | | 0.002 | | | |
| Depression | 4.02 (3.18) | 4.54 (3.32) |  | 6.0 | | <.001 | | | | 0.002 | | | |
| Self-perceived health (Chinese Health Questionnaire) | 1.24 (1.75) | 1.36 (1.82) |  | 2.4 | | 0.016 | | | | 0.002 | | | |
| Behavioral problems (Child Behavior Checklist) | | |  |  | |  | |  | | | |  |  |
| Aggressive behavior | 3.53 (4.28) | 4.60 (5.03) |  | 8.7 | | <.001 | | | | 0.002 | | | |
| Anxious/depressed | 2.48 (3.30) | 3.13 (3.82) |  | 7.0 | | <.001 | | | | 0.002 | | | |
| Attention problems | 2.94 (3.12) | 3.60 (3.48) |  | 7.6 | | <.001 | | | | 0.002 | | | |
| Delinquent behavior | 1.10 (1.95) | 1.43 (2.16) |  | 6.2 | | <.001 | | | | 0.002 | | | |
| Social problems | 1.78 (2.04) | 2.10 (2.25) |  | 5.7 | | <.001 | | | | 0.002 | | | |
| Somatic complaints | 0.89 (1.79) | 1.17 (2.10) |  | 5.3 | | <.001 | | | | 0.002 | | | |
| Thought problems | 0.79 (1.32) | 1.13 (1.62) |  | 8.7 | | <.001 | | | | 0.002 | | | |
| Withdrawn | 1.72 (2.11) | 2.18 (2.47) |  | 7.6 | | <.001 | | | | 0.002 | | | |
| Social school function (SAICA) | |  |  |  |  | |  | | | |  |  |  |
| School function | 1.50 (1.49) | 1.50 (1.49) |  | 0.1 | | 0.908 | | | | 0.908 | | | |
| Peer interaction | 1.53 (1.52) | 1.54 (1.52) |  | 0.5 | | 0.620 | | | | 0.698 | | | |
| Home behavior | 1.29 (1.28) | 1.34 (1.33) |  | 6.3 | | <.001 | | | | 0.002 | | | |

Note: Among 4816 children who were allowed to undergo the clinical interview, only 4240 parents completed the parent questionnaires.

Adjusted p value was corrected using the false discovery rate method.

Family APGAR = Family Adaptation, Partnership, Growth, Affection, and Resolve; K-SADS-E = Schedule for Affective Disorders and Schizophrenia for School-Age Children-Epidemiologic version;

SAICA = Social Adjustment Inventory for Children and Adolescents.

# Supplementary Table S4. The weighted and 6-month weight prevalence of diagnostic distribution of DSM-5 mental disorders in Taiwan's National Epidemiological Study of Child Mental Disorders

|  | Weighted prevalence | | | | | |  | IPCW-adjusted prevalence | | | | Prevalence ratio (95% CI) | |  |
| --- | --- | --- | --- | --- | --- | --- | --- | --- | --- | --- | --- | --- | --- | --- |
|  | Lifetime | | | 6-month | | |  | Lifetime | | 6-month | | Lifetime | 6-month |  |
| DSM-5 diagnoses | N | _wt_% | 95% CI | N | _wt_% | 95% CI |  | _wt_% | 95% CI | _wt_% | 95% CI |  |  |  |
| Neurodevelopmental disorders |  |  |  |  |  |  |  |  |  |  |  |  |  |  |
| Autism spectrum disorder^a^ | 52 | 1.0 | 0.6-1.5 | - | - | - |  | 1.0 | 0.6-1.5 | - | - | 1.00 (0.67-1.49) | - |  |
| ADHD^b^ | 487 | 10.5 | 9.3-11.7 | 412 | 9.0 | 7.6-10.4 |  | 10.1 | 8.9-11.3 | 8.7 | 7.3-10.1 | 1.04 (0.92-1.17) | 1.03 (0.91-1.18) |  |
| Tic disorders^c^ | 151 | 2.7 | 1.9-3.4 | 126 | 2.3 | 1.6-2.9 |  | 2.6 | 2.0-3.4 | 2.1 | 1.4-2.7 | 1.04 (0.82-1.32) | 1.10 (0.84-1.43) |  |
| DICCD |  |  |  |  |  |  |  |  |  |  |  |  |  |  |
| Oppositional defiant disorder | 97 | 2.1 | 1.5-2.7 | 76 | 1.6 | 1.1-2.2 |  | 2.0 | 1.4-2.6 | 1.5 | 0.9-2.1 | 1.05 (0.80-1.38) | 1.07 (0.78-1.47) |  |
| Conduct disorder | 15 | 0.5 | 0.1-0.9 | 9 | 0.1 | 0.0-0.3 |  | 0.5 | 0.1-0.9 | 0.1 | 0.0-0.3 | 1.00 (0.57-1.76) | 1.00 (0.28-3.53) |  |
| Intermittent explosive disorder | 17 | 0.2 | 0.0-0.4 | 10 | 0.1 | 0.0-0.2 |  | 0.2 | 0.0-0.4 | 0.1 | 0.0-0.3 | 1.00 (0.41-2.44) | 2.00 (0.67-5.97) |  |
| Depressive disorders |  |  |  |  |  |  |  |  |  |  |  |  |  |  |
| Major depressive disorder | 79 | 1.4 | 0.8-2.0 | 24 | 0.5 | 0.1-0.9 |  | 1.7 | 0.7-2.7 | 0.7 | 0.0-1.5 | 0.82 (0.60-1.13) | 0.71 (0.42-1.20) |  |
| Persistent depressive disorder | 28 | 0.5 | 0.1-0.9 | 15 | 0.1 | 0.0-0.2 |  | 0.8 | 0.0-1.6 | 0.2 | 0.0-0.4 | 0.63 (0.38-1.04) | 0.50 (0.17-1.49) |  |
| DMDD | 14 | 0.3 | 0.1-0.5 | 12 | 0.3 | 0.0-0.5 |  | 0.3 | 0.1-0.5 | 0.2 | 0.0-0.4 | 1.00 (0.48-2.07) | 1.50 (0.66-3.39) |  |
| Anxiety disorders |  |  |  |  |  |  |  |  |  |  |  |  |  |  |
| Any anxiety disorder | 702 | 15.1 | 12.9-17.3 | 550 | 11.8 | 10.1-13.5 |  | 15.2 | 12.7-17.7 | 12.0 | 10.0-14.0 | 0.99 (0.90-1.09) | 1.13 (1.01-1.25) |  |
| Generalized anxiety disorder | 33 | 0.9 | 0.3-1.5 | 30 | 0.7 | 0.2-1.1 |  | 0.9 | 0.3-1.5 | 0.7 | 0.3-1.1 | 1.00 (0.66-1.52) | 0.29 (0.14-0.58) |  |
| Social anxiety disorder | 154 | 3.6 | 2.8-4.4 | 137 | 3.0 | 2.1-3.6 |  | 3.6 | 2.6-4.6 | 2.7 | 1.9-3.5 | 1.00 (0.81-1.23) | 1.11 (0.88-1.40) |  |
| Specific phobia disorder | 412 | 8.7 | 6.9-10.5 | 366 | 7.7 | 6.2-9.1 |  | 8.7 | 6.9-10.5 | 7.7 | 6.1-9.3 | 1.00 (0.88-1.14) | 1.00 (0.87-1.15) |  |
| Separation anxiety disorder | 178 | 4.2 | 3.4-5.0 | 59 | 1.9 | 0.9-2.7 |  | 4.4 | 3.2-5.6 | 2.2 | 0.8-3.6 | 0.95 (0.79-1.15) | 0.86 (0.65-1.14) |  |
| Panic disorder | 19 | 0.4 | 0.0-0.8 | 8 | 0.1 | 0.0-0.2 |  | 0.4 | 0.0-0.8 | 0.1 | 0.0-0.2 | 1.00 (0.53-1.88) | 1.00 (0.28-3.53) |  |
| Agoraphobia | 13 | 0.4 | 0.0-0.8 | 13 | 0.3 | 0.0-0.8 |  | 0.4 | 0.0-0.8 | 0.4 | 0.0-0.8 | 1.00 (0.53-1.88) | 0.75 (0.38-1.48) |  |
| Feeding and eating disorders |  |  |  |  |  |  |  |  |  |  |  |  |  |  |
| Anorexia nervosa | 4 | 0.2 | 0.0-0.4 | 3 | 0.2 | 0.1-0.5 |  | 0.2 | 0.0-0.4 | 0.2 | 0.0-0.4 | 1.00 (0.41-2.44) | 1.00 (0.41-2.44) |  |
| ARFID | 40 | 0.5 | 0.3-0.7 | 22 | 0.3 | 0.1-0.5 |  | 0.5 | 0.3-0.7 | 0.3 | 0.1-0.5 | 1.00 (0.57-1.76) | 1.00 (0.48-2.07) |  |
| Obsessive-compulsive disorder | 47 | 1.2 | 0.4-2.0 | 26 | 0.6 | 0.1-1.0 |  | 1.4 | 0.2-2.6 | 0.8 | 0.0-1.6 | 0.86 (0.60-1.22) | 1.00 (0.64-1.56) |  |
| Schizophrenia | 4 | 0.1 | 0.0-0.2 | 4 | 0.1 | 0.1-1.0 |  | 0.1 | 0.0-0.3 | 0.1 | 0.0-0.3 | 1.00 (0.28-3.53) | 1.00 (0.28-3.53) |  |
| Gender dysphoria | 14 | 0.3 | 0.1-0.5 | 13 | 0.1 | 0.0-0.2 |  | 0.3 | 0.1-0.5 | 0.3 | 0.1-0.5 | 1.00 (0.48-2.07) | 0.33 (0.12-0.93) |  |
| Reactive attachment disorder | 4 | 0.1 | 0.0-0.2 | 4 | 0.1 | 0.1-0.5 |  | 0.1 | 0.0-0.2 | 0.1 | 0.0-0.2 | 1.00 (0.28-3.53) | 1.00 (0.28-3.53) |  |
| Post-traumatic stress disorder | 7 | 0.2 | 0.1-0.3 | 0 | 0.0 | 0.0-0.0 |  | 0.1 | 0.0-0.2 | 0.0 | 0.0-0.0 | 2.0 (0.72-5.88) | - |  |
| Dissociative identity disorder | 3 | 0.2 | 0.0-0.4 | 1 | 0.1 | 0.0-0.2 |  | 0.4 | 0.0-0.8 | 0.2 | 0.0-0.4 | 0.50 (0.23-1.08) | 0.50 (0.17-1.49) |  |
| Any sleep disorders^c^ | 473 | 11.7 | 9.0-14.4 | 274 | 6.2 | 5.0-7.4 |  | 12.0 | 9.1-14.9 | 6.2 | 4.8-7.6 | 0.98 (0.87-1.09) | 1.00 (0.86-1.17) |  |
| Insomnia disorder | 105 | 2.1 | 1.2-2.9 | 92 | 1.8 | 0.9-2.7 |  | 2.2 | 1.3-3.0 | 1.8 | 0.9-2.7 | 0.95 (0.73-1.25) | 1.00 (0.74-1.34) |  |
| Hypersomnolence disorder | 3 | 0.1 | 0.0-0.3 | 3 | 0.1 | 0.0-0.3 |  | 0.1 | 0.0-0.3 | 0.1 | 0.0-0.3 | 1.00 (0.28-3.53) | 1.00 (0.28-3.53) |  |
| Circadian rhythm sleep-wake disorders | 2 | 0.1 | 0.0-0.2 | 2 | 0.1 | 0.0-0.2 |  | 0.1 | 0.0-0.2 | 0.1 | 0.0-0.2 | 1.00 (0.28-3.53) | 1.00 (0.28-3.53) |  |
| Nightmare disorder | 337 | 8.6 | 6.1-11.1 | 171 | 4.0 | 2.8-5.2 |  | 8.9 | 6.3-11.4 | 4.1 | 2.9-5.3 | 0.97 (0.85-1.10) | 0.98 (0.80-1.18) |  |
| NREM sleep arousal disorders-sleepwalking | 63 | 1.4 | 1.0-1.8 | 23 | 0.4 | 0.2-0.6 |  | 1.4 | 1.0-1.8 | 0.4 | 0.2-0.6 | 1.00 (0.72-1.40) | 1.00 (0.53-1.88) |  |
| NREM sleep arousal disorders-sleep terror | 27 | 0.4 | 0.2-0.6 | 13 | 0.1 | 0.0-0.2 |  | 0.4 | 0.2-0.6 | 0.1 | 0.0-0.2 | 1.00 (0.53-1.88) | 1.00 (0.28-3.53) |  |
| Restless legs syndrome | 8 | 0.2 | 0.0-0.4 | 8 | 0.2 | 0.0-0.4 |  | 0.3 | 0.0-0.7 | 0.3 | 0.0-0.7 | 0.67 (0.30-1.51) | 0.67 (0.30-1.51) |  |
| Any class^d^ |  |  |  |  |  |  |  |  |  |  |  |  |  |  |
| 1 class | 1499 | 32.5 | 28.9-36.1 | 1187 | 25.7 | 22.8-28.6 |  | 31.6 | 28.1-35.3 | 25.0 | 22.1-27.9 | 0.97 (0.92-1.03) | 0.97 (0.91-1.04) |  |
| 2 class | 611 | 13.2 | 11.4-15.1 | 416 | 8.6 | 7.1-10.2 |  | 12.9 | 10.5-14.2 | 8.3 | 6.8-9.9 | 0.98 (0.88-1.08) | 0.97 (0.85-1.10) |  |
| 3 class | 244 | 5.7 | 4.2-7.1 | 149 | 3.2 | 2.4-3.9 |  | 5.6 | 4.1-7.0 | 3.1 | 2.3-3.8 | 0.98 (0.83-1.16) | 0.97 (0.78-1.21) |  |
| Suicide-related problems |  |  |  |  |  |  |  |  |  |  |  |  |  |  |
| Suicidal ideation | 377 | 7.9 | 6.1-9.7 | 163 | 3.1 | 2.3-3.9 |  | 8.2 | 6.0-10.4 | 3.1 | 2.3-3.9 | 0.96 (0.84-1.10) | 1.00 (0.80-1.25) |  |
| Suicidal plan | 153 | 3.6 | 2.2-5.0 | 77 | 1.6 | 0.6-2.6 |  | 3.6 | 2.2-5.0 | 1.7 | 0.7-2.7 | 1.00 (0.81-1.23) | 0.94 (0.69-1.28) |  |
| Suicidal attempt | 43 | 0.7 | 0.3-1.1 | 19 | 0.3 | 0.1-0.5 |  | 0.7 | 0.3-1.1 | 0.3 | 0.1-0.5 | 1.00 (0.62-1.61) | 1.00 (0.48-2.07) |  |

IPCW-adjusted prevalence serves as the reference group.

ADHD = attention-deficit hyperactivity disorder; ARFID = avoidant/restrictive food intake disorder; DMDD = disruptive mood dysregulation disorder; DMDD = disruptive mood dysregulation disorder; DICCD = disruptive, impulse-control, and conduct disorders; NREM = non-rapid eye movement; IPCW = inverse probability censoring weighting. ^a^Autism spectrum disorder is considered a lifelong disorder; only lifetime weighted prevalence was reported. ^b^Attention-deficit/hyperactivity disorder includes three subtypes: predominantly inattentive, predominantly hyperactive-impulsive, and combined type. ^b^Tic disorders include Tourette's disorder, persistent and provisional motor disorder, or vocal tic disorder. ^c^Any sleep disorders include insomnia, hypersomnolence, sleep apnea, circadian rhythm sleep-wake disorders, non-rapid eye movement sleep arousal disorders (sleepwalking and sleep terror), nightmare, and restless legs syndrome. ^d^Any mental disorders do not include suicide-related problems.

**References**

**Cheng T-A Williams P** (1986). The design and development of a screening questionnaire (CHQ) for use in community studies of mental disorders in Taiwan. *Psychological Medicine* **16**, 415-422.

**Gau SS-F, Lai M-C, Chiu Y-N, Liu C-T, Lee M-B Hwu H-G** (2009). Individual and family correlates for cigarette smoking among Taiwanese college students. *Comprehensive Psychiatry* **50**, 276-285.

**Gau SS-F, Shen H-Y, Chou M-C, Tang C-S, Chiu Y-N Gau C-S** (2006). Determinants of adherence to methylphenidate and the impact of poor adherence on maternal and family measures. *Journal of Child and Adolescent Psychopharmacology* **16**, 286-297.

**Liu C, Hung Y, Chuang Y, Chen Y, Weng W, Liu J Liang K** (2006). Incorporating development stratification of Taiwan townships into sampling design of large scale health interview survey. *Journal of Health Management* **4**, 1-22.

**Shang CY, Gau SSF Soong WT** (2006). Association between childhood sleep problems and perinatal factors, parental mental distress and behavioral problems. *Journal of Sleep Research* **15**, 63-73.

**Yeh CB, Gau SSF, Kessler RC Wu YY** (2008). Psychometric properties of the Chinese version of the adult ADHD Self‐report Scale. *International Journal of Methods in Psychiatric Research* **17**, 45-54.
